# Associations between Frequency of Dairy Intake with Body Composition and Excess Adiposity in Preschool Children from Poland

**DOI:** 10.3390/ijerph19031140

**Published:** 2022-01-20

**Authors:** Piotr Matłosz, Justyna Wyszyńska, Wojciech Czarny, Artur Mazur, Jarosław Herbert

**Affiliations:** 1 Institute of Physical Culture Sciences, Medical College, University of Rzeszów, 35-326 Rzeszów, Poland; wczarny@ur.edu.pl (W.C.); jherbert@ur.edu.pl (J.H.); 2 Institute of Health Sciences, Medical College, University of Rzeszów, 35-310 Rzeszów, Poland; justyna.wyszynska@onet.pl; 3 Institute of Medical Sciences, Medical College, University of Rzeszów, 35-959 Rzeszów, Poland; drmazur@poczta.onet.pl

**Keywords:** adiposity, body fat, cheese, dairy products, fat-free mass, milk, yoghurt

## Abstract

Evidence suggests there has been a decrease in childhood dairy consumption. There is a need for further studies to evaluate the types of dairy products in relation to the risk of obesity in pediatric population. The aim of the study was to determine the associations between the frequency of consumption of different types of dairy products and body composition and excess adiposity in preschool children from Poland. A cross-sectional study of 1172 children aged 5–6 years was conducted using a bioelectrical impedance analysis for body composition estimation and a modified food frequency questionnaire (FFQ-6) to assess the habitual diets of the participants. Among the analyzed dairy products, milk was consumed most often, followed by fruit yoghurts, yellow cheese, and cottage cheese, and natural yoghurt was the least common. Participants that consumed fruit or natural yoghurt more frequently had higher adipose tissue content. The logistic regression analysis by the method of forward selection showed that more frequent consumption of fruit yoghurt significantly increased the risk of excess adiposity among the total sample (OR = 1.20, *p* < 0.001). This study revealed that higher consumption of fruit yoghurt could be significant determinant of excess adiposity in Polish preschool children.

## 1. Introduction

Childhood obesity is major public health concern and may negatively affect the immediate health of the child. While the cause of excess weight is multifactorial, changes in diet and decreased level of physical activity have been proposed as contributing factors to the obesity epidemic [1]. Evidence suggests a decrease in dairy consumption coincides with the rise in obesity prevalence in pediatric population [2]. A large percentage of children do not consume the recommended amounts of low-fat and fat-free dairy products, fruits, vegetables, and whole grains, which results in low intakes of calcium, potassium, fiber, magnesium, and vitamin E [3].

Spence et al. [3], in their study analyzing the role of dairy products in healthy weight and body composition in children and adolescents, concluded that the available studies indicated that the consumption of milk and milk products do not have a negative effect on body weight or body composition in pediatric population. However, the authors noted the need for additional research to better understand the relationship between dairy food consumption and obesity prevalence in school-age children. A recent meta-analysis on the effects of dairy consumption on body composition and bone properties in youth showed that systematic consumption of dairy products may benefit bone structure and development, but an effect on body composition or body size in pediatric population was not indicated [4]. Another meta-analysis of long-term association between dairy consumption and risk of childhood obesity, comprising 46,011 children and adolescents, suggested that dairy intake is negatively and longitudinally related with the risk of excess weight in children and adolescents. The authors emphasized the need for further research to assess the relationship between the type of dairy products and the risk of excess body weight in children [5]. Therefore, the aim of the study was to determine the associations between the frequency of consumption of different types of dairy products and body composition and excess adiposity in preschool children from Poland.

## 2. Materials and Methods

### 2.1. Study Design and Study Sample

Written informed consent was obtained from parents or legal guardians prior to participation in the study. The study was approved by the Bioethics Committee at the Medical Department of the University of Rzeszów, Poland, and it was conducted in accordance with ethical standards laid down in an appropriate version of the Declaration of Helsinki. The study was conducted from October to November in each year from 2018 to 2019. The details of the study design and a partial analysis have been reported elsewhere [6].

### 2.2. Anthropometric Measurements

Children’s body weight and height were measured, using standard protocol and equipment, which was calibrated before and during the period of data collection. A portable stadiometer (HR-200, Tanita, Tokyo, Japan) was used to assess body height. Body weight was measured with a body composition analyzer (BC-420 MA, Tanita, Tokyo, Japan). Individual body mass index (BMI) was calculated as body mass (kg) divided by height (m) squared.

### 2.3. Body Composition

The body composition was measured using a bioelectrical impedance analysis (BIA) method by body composition analyzer (BC-420 MA, Tanita, Tokyo, Japan). The participants’ parents were instructed to ensure their child did not consume any meals or drinks for at least 8 h before the examination [7]. For the aim of this study, the participants were classified as (1) “no excess adiposity”—body fat percentage (BFP) < 85th percentile and (2) “excess adiposity”—BFP ≥ 85th percentile. For this purpose, BFP charts by age and sex were used [8].

### 2.4. Food Frequency Questionnaire (FFQ)

To assess the habitual diets of the participants, the modified food frequency questionnaire (FFQ-6) was used, which was self-reported by the parents/caregivers [9]. Noor Hafizah et al. [10] found good reliability and acceptable validity of a food frequency questionnaire reported by caregivers of preschool children compared to a 3-day diet record. The questionnaire included questions about the frequency of consumption of selected dairy products during the previous 12 months, i.e., milk, fruit yoghurt, natural yoghurt, cottage cheese and yellow cheese. The respondents had a choice of six categories of food consumption frequency, with higher scores indicating more frequent food intake: (1) never or almost never, (2) once a month or less, (3) several times a month, (4) a few times a week, (5) daily, and (6) several times a day.

### 2.5. Statistical Analysis

Statistical analysis was performed using the SPSS 25 software (IBM, North Harbour, UK). The normality of distribution of continuous variables was tested with a Shapiro–Wilk test. Continuous variables were presented as mean (±SD), and categorical data were presented as n (%). The Mann–Whitney test was used to present a statistically significant difference between boys and girls. The associations between the consumption of dairy products and body composition was determined using the Spearman’s rho correlation coefficient. Logistic regression analysis by the method of forward selection (likelihood ratios) was used to identify factors associated with an excess adiposity. The level of statistical significance was adopted at *p* < 0.05.

## 3. Results

The study group consisted of 1172 children (48.8% girls). The general characteristics of the study population, according to sex, are shown in Table 1. Boys were taller than girls by 1.4 cm. The content of adipose tissue was significantly higher in girls than in boys, by almost 1%. Muscle mass and fat-free mass (FFM) were significantly higher in boys than in girls, by 0.47% and 0.95%, respectively. No significant differences were found with regard to weight, BMI, and muscle mass between girls and boys.

Table 2 shows the distribution of frequency of consumption of dairy products in children with relation to sex. Among the analyzed dairy products, milk was consumed most often, followed by fruit yoghurts, yellow cheese, and cottage cheese, with natural yoghurt the least common. No significant difference in frequency of consumption of dairy products among girls and boys was found (*p* < 0.05).

In Table 3, associations between frequency of consumption of dairy products and body composition among the study population are presented. The analysis of the results showed a positive association between the frequency of fruit yoghurt consumption and BFP in total sample and girls, and negative correlation with muscle mass, FFM, and TBW. However, all the associations were weak. Similar associations were found for natural yoghurt, but only in boys and total sample. A positive association between the frequency of natural yoghurt consumption and BFP, and negative correlation with muscle mass, FFM, and TBW was observed. The above associations were rather negligible (rho < 0.1).

The associations between the frequency of dairy products consumption and the excess BFP prevalence are presented in Table 4. More frequent consumption of milk, fruit yoghurt, natural yoghurt, and yellow cheese was significantly associated with excess BFP in total sample. Girls and boys with excess BFP consumed fruit yoghurt more frequently. Moreover, more frequent consumption of milk among girls and natural yoghurt among boys was associated with excess adiposity.

Table 5 presents a logistic regression analysis by the method of forward selection to identify factors associated with an excess BFP. More frequent consumption of fruit yoghurt significantly increased the risk of excess adiposity among total sample (OR = 1.20, *p* < 0.001). Among girls, excess BPF was associated with more frequent consumption of milk and fruit yoghurt (OR = 1.21, *p* < 0.05). Among boys, excess BPF was also associated with more frequent consumption of fruit yoghurt (OR = 1.17, *p* = 0.025) and natural yoghurt (OR = 1.15, *p* = 0.023).

## 4. Discussion

To the best of our knowledge, this is the first study to evaluate the associations between frequency of dairy products consumption and body composition and excess adiposity in preschool children from Poland. Overall, our findings suggest that among the analyzed dairy products, milk and fruit yoghurt were consumed most often, both in girls and in boys. Yellow cheese and cottage cheese were eaten less frequently, and natural yoghurt was the least frequently consumed. No difference in frequency of consumption of dairy products among girls and boys was found. Slightly different results are provided in the study of Abreu et al. [11], who aimed to examine the association between dairy product intake and BMI and BFP in adolescents from the Azorean Archipelago, Portugal. The authors found that boys’ diets were significantly higher in energy, total fat, total dairy, milk, and calcium intake and lower in carbohydrates and dietary fiber, compared with girls diets. There was no significant difference between boys and girls with regard to protein, sugar, yogurt, and cheese intake.

Our results showed a positive association between the frequency of fruit yoghurt consumption and BFP in total sample and girls; however, these associations were weak (rho, 0.10–0.39) [12]. Moreover, a positive association between the frequency of natural yoghurt consumption and BF was observed in boys and total sample; however, these associations are negligible (rho < 0.1) [12]. The literature related to dairy product consumption, especially different types of dairy, and excess weight in pediatric population is limited and inconsistent. Evidence from cross-sectional studies suggested that dairy consumption is associated with lower BMI and BFP in children and adolescents [13,14,15,16]. Results from the National Health and Nutrition Examination Survey (NHANES 2005–2008) showed that yogurt and dairy intake were connected with greater calcium and vitamin D intakes, and that higher yogurt, dairy, calcium, and vitamin D intake were associated with lower BFP in US children [15]. Abreu et al. [11] showed that milk intake was negatively and significantly associated with BMI and BFP, but only in girls. In another study, Abreu et al. [17] indicated that adolescents with high milk consumption had lower risk of abdominal obesity, compared with adolescents with low milk consumption. Nevertheless, prospective studies have yielded inconsistent results. A study of 852 preschool-aged children in US showed that milk consumption at age 2, whether full or low-fat, was not associated with risk of overweight at age 3. Neither total milk nor total dairy consumption at age 2 was related with BMI or prevalence of overweight at age 3 [18]. A recent prospective study of healthy children aged from 9 months to 8 years showed that higher cow’s milk fat consumption was related with lower zBMI and lower odds of excess weight. Each 1% increase in cow’s milk fat intake was connected with a 0.05 lower zBMI score. Moreover, participants who consumed whole milk had 16% lower odds of overweight (OR  =  0.84) and 18% lower odds of obesity (OR  =  0.82), compared to children who consumed reduced-fat (0.1–2%) milk [19].

Controlled intervention studies have also examined the associations between dairy and/or milk consumption and BPF or body weight. Calleja et al. [20], in a randomized controlled trial, determined whether increased dairy product intake, as part of a lifestyle modification program featuring exercise training and dietary guidance, promotes favorable body composition changes in adolescent girls with excess weight. Two experimental groups: recommended dairy (4 servings/day of dairy foods) and low dairy (0–2 servings/day of dairy foods) participated in a 12-week, eucaloric, lifestyle modification intervention consisting of mixed-mode exercise (3×/week) and nutritional counseling. After intervention, weight did not significantly change in any group. Recommended dairy significantly decreased fat mass and increased lean mass compared to low dairy group and no-intervention control group. Low dairy also significantly decreased fat mass and increased lean mass more than control group did. The authors concluded that the inclusion of dairy foods in the diet of adolescent females with excess weight favorably improves body composition in the absence of weight loss. St-Onge et al. [21], in randomized controlled trial, also examined whether high milk (4 servings/day) intake, compared to low milk (1 serving/day) consumption, was associated with greater weight loss in overweight children during the 16-week healthy-eating diet intervention. The authors found no significant differences between the groups in weight loss.

Our results showed that more frequent consumption of milk, fruit yoghurt, natural yoghurt, and yellow cheese was significantly associated with excess BFP in total sample of preschool children. However, logistic regression analysis showed that the strongest predictor of excess adiposity in boys and girls was higher consumption of fruit yoghurt. This relationship may be explained by the fact that fruit yoghurts contain high amounts of carbohydrates (total sugars), which promote the development of obesity [22]. In the literature, the majority of studies have examined milk intake while only a few have examined other dairy products. Abreu et al. [11] showed no significant association between yogurt and cheese intake with BMI and BFP; however, this study was conducted on a different population at a different age. Discrepancies in the findings of existing studies could be explained by differences in study design, methods for assessing diet and body composition, and adjustment of potentially confounding factors, and/or due to the complexity of interactions between nutrients in humans.

To comprehensively interpret the results, the particular age of the subjects should also be considered. The present study was conducted in kindergartens; therefore, during weekdays it could be assumed that half to two-thirds of daily nutrient needs were provided by the kindergarten staff, leaving one-third to one-half of daily intake to be chosen by parents. The same could be applied to meal times. Since the kindergarten menu in most cases is prepared under the supervision and guidance of dietitians, it seems to be particularly important to ensure an adequate level of nutritional education for parents. In order to prevent overweight and obesity at a later age, in addition to teaching preschool children to make appropriate nutrition choices, children should also be encouraged to maintain a high level of physical activity and minimize sedentary behaviors. This becomes especially important in the later years, when children start attending school and decide more about their own nutrition. As the child grows older, external factors such as the environment of peers, media coverage, stressful situations related to school and family environment, and eating habits in the family will have an increasing influence on the child’s nutritional decisions.

The potential mechanism underlying any relationship between dairy products consumption and adiposity remains unclear. Several mechanisms were proposed to explain how dairy might affect energy balance and body composition. Dairy products are a significant source of calcium, vitamin D, and protein that may promote lower adiposity [23,24]. It has also been suggested that milk is rich in bioactive peptides that may act independently of calcium to modulate body fat accumulation [25]. Milk also contains many bovine hormones and growth factors identical to those found in humans [26]. While many hormones are digested or metabolized, those that are absorbed intact may have a potential impact on growth and metabolism [26].Moreover, evidence suggested that the replacement of milk intake with sugar sweetened beverages has contributed to an increase in adiposity in children and adolescents [27,28].

Due to the cross-sectional nature of the study and limited data, it is difficult to unambiguously explain the potential mechanism that could affect the relationships between the consumption of dairy products and body fat in the studied group. The observed differences in significant correlations between the frequency of fruit yoghurt consumption and BFP according to sex could be the effect of several additional factors that were not assessed in the present study, such as differences in energy expenditure (physical activity and sedentary behaviors), lifestyle factors, individual metabolism and other predispositions to obesity, as well as caloric intake from non-dairy products or the combination of certain products in the diet. The lack of these data should be considered an important limitation of the present study, as all of these factors could potentially affect the results.

Our study has several strengths and limitations. A limitation of this study is the cross-sectional design, meaning the causal pathways underlying the observed associations could not be examined. Moreover, we cannot be certain that confounding factors, such as intake of other food products, level of physical activity, or genetic factors, have not influenced our results. There were insufficient data to calculate total daily energy, which would have improved our understanding of the mechanism underlying the observed relationship. Data collection was carried out in the fall; therefore, environmental factors could also potentially affect the physical activity patterns of children, which may influence body composition in addition to dairy intake. Although measures of both caloric intake in the diet (also from non-dairy products or the combination of certain products) and energy expenditure (the level of physical activity and sedentary lifestyle) would have enriched our ability to draw conclusions from the results, the lack of those measures does not negate the relevance of this study to public health.

The study, however, benefits from a number of strengths, including a relatively large sample size and a focus on early childhood. That is an important time frame that should be targeted by preventive strategies intended to reduce body fat and consequently, decrease the risk of noncommunicable diseases. Moreover, body composition was measured by using the BIA method that has demonstrated excellent test–retest reliability, moderately strong absolute agreement with DEXA, and high specificity for overfat and obese classification [7].

Future research should include a greater emphasis on longitudinal studies to determine the long-term impact on weight and adiposity of inclusion of dairy products in children and adolescent. It is also important to examine the mechanisms by which dairy impacts weight and adiposity in order to provide a physiological understanding.

## 5. Conclusions

Dairy products are usually the core of a balanced diet for children at an early age. Evidence-based and thoughtful directions for caregivers regarding dairy products intake could reinforce a harmonious and healthy development of adolescents. The results of the present study suggest that higher consumption of fruit yoghurt in Polish preschool children could be a factor that contributes to excess adiposity in this population. However, when interpreting these results, it should be taken into account that the potential mechanism by which the consumption of dairy products affects body fat is very complex and depends on many other factors.

## Figures and Tables

**Table 1:** General characteristics of the study population.

Variables	Girls		Boys		Total Sample		*p*
	Mean	SD	Mean	SD	Mean	SD	
Age (years)	5.52	0.50	5.53	0.50	5.52	0.50	0.547
Height (cm)	116.09	5.54	117.49	5.94	116.80	5.79	**<0.001**
Weight (kg)	21.27	3.72	21.68	3.94	21.48	3.84	0.066
BMI (kg/m^2^)	15.69	1.79	15.61	1.84	15.65	1.82	0.253
BFP	20.66	4.97	19.71	4.16	20.17	4.60	**<0.001**
Muscle mass (%)	75.10	4.60	75.57	3.55	75.34	4.10	0.050
FFM (%)	79.34	4.97	80.29	4.16	79.83	4.60	**<0.001**
TBW (%)	58.10	3.56	58.87	2.81	58.49	3.22	**<0.001**

BFP—body fat percentage; BMI –body mass index; FFM—fat-free mass; TBW—total body water; *p*-value represents the differences between boys and girls; significant associations are highlighted in bold.

**Table 2:** The frequency of dairy products consumption among the study population.

Dairy Product				Milk	Fruit Yoghurt	Natural Yoghurt	Cottage Cheese	Yellow Cheese
Frequency	Girls	Never or almost never	n	16	45	217	60	79
			%	2.8	7.9	37.9	10.5	13.8
		Once a month or less	n	5	33	65	40	19
			%	0.9	5.8	11.4	7.0	3.3
		Several times a month	n	29	72	104	116	73
			%	5.1	12.6	18.2	20.3	12.8
		Few times a week	n	178	253	139	281	290
			%	31.1	44.2	24.3	49.1	50.7
		Daily	n	250	160	45	70	102
			%	43.7	28.0	7.9	12.2	17.8
		Several times a day	n	94	9	2	5	9
			%	16.4	1.6	0.3	0.9	1.6
	Boys	Never or almost never	n	19	59	244	77	86
			%	3.2	9.8	40.7	12.8	14.3
		Once a month or less	n	9	25	69	32	19
			%	1.5	4.2	11.5	5.3	3.2
		Several times a month	n	18	82	106	118	66
			%	3.0	13.7	17.7	19.7	11.0
		Few times a week	n	194	279	146	298	288
			%	32.3	46.5	24.3	49.7	48.0
		Daily	n	274	141	33	68	135
			%	45.7	23.5	5.5	11.3	22.5
		Several times a day	n	86	14	2	7	6
			%	14.3	2.3	0.3	1.2	1.0
	Girls		mean	4.61	3.83	2.54	3.48	3.60
			SD	1.03	1.18	1.42	1.15	1.26
	Boys		mean	4.59	3.77	2.44	3.45	3.64
			SD	1.03	1.21	1.39	1.19	1.29
	Total		mean	4.60	3.80	2.49	3.47	3.62
			SD	1.03	1.19	1.40	1.17	1.28
*p*				0.739	0.256	0.221	0.755	0.275

*p*-value represents the differences between boys and girls.

**Table 3:** Associations between dairy consumption and body composition among the study population.

Body Composition		Milk	Fruit Yoghurt	Natural Yoghurt	Cottage Cheese	Yellow Cheese
Total sample (*n* = 1172)						
BFP	rho	0.035	0.114	0.081	−0.007	0.041
	*p*	0.231	**<0.001**	**0.005**	0.812	0.158
Muscle mass (%)	rho	−0.033	−0.110	−0.077	0.010	−0.039
	*p*	0.256	**<0.001**	**0.008**	0.733	0.181
FFM (%)	rho	−0.035	−0.114	−0.081	0.007	−0.041
	*p*	0.231	**<0.001**	**0.005**	0.812	0.158
TBW (%)	rho	−0.031	−0.114	−0.081	0.011	−0.038
	*p*	0.294	**<0.001**	**0.006**	0.704	0.188
Girls (*n* = 572)						
BFP	rho	0.081	0.136	0.069	−0.029	0.024
	*p*	0.053	**0.001**	0.101	0.485	0.568
Muscle mass (%)	rho	−0.081	−0.135	−0.068	0.031	−0.023
	*p*	0.052	**0.001**	0.105	0.459	0.589
FFM (%)	rho	−0.081	−0.136	−0.069	0.029	−0.024
	*p*	0.053	**0.001**	0.101	0.485	0.568
TBW (%)	rho	−0.078	−0.136	−0.072	0.034	−0.020
	*p*	0.062	**0.001**	0.087	0.423	0.636
Boys (*n* = 600)						
BFP	rho	−0.018	0.073	0.098	0.022	0.065
	*p*	0.651	0.072	**0.016**	0.585	0.110
Muscle mass (%)	rho	0.024	−0.071	−0.093	−0.017	−0.058
	*p*	0.552	0.081	**0.022**	0.685	0.157
FFM (%)	rho	0.018	−0.073	−0.098	−0.022	−0.065
	*p*	0.651	0.072	**0.016**	0.585	0.110
TBW (%)	rho	0.022	−0.074	−0.094	−0.019	−0.065
	*p*	0.583	0.072	**0.022**	0.639	0.112

BFP—body fat percentage; FFM—fat-free mass; *p*—statistical significance; rho—Spearman’s correlation coefficient; TBW—total body water; significant associations are highlighted in bold.

**Table 4:** Associations between the frequency of dairy products consumption and the excess BFP.

BFP Classification					
Dairy Product	No Excess BFP		Excess BFP		*p*
	Mean	SD	Mean	SD	
Total sample (*n* = 1172)					
Milk	4.54	1.07	4.68	0.97	**0.049**
Fruit yoghurt	3.69	1.24	3.95	1.11	**0.001**
Natural yoghurt	2.41	1.39	2.59	1.41	**0.028**
Cottage cheese	3.45	1.15	3.48	1.19	0.644
Yellow cheese	3.56	1.28	3.71	1.26	**0.025**
Girls (*n* = 572)					
Milk	4.53	1.08	4.75	0.93	**0.019**
Fruit yoghurt	3.73	1.20	4.01	1.11	**0.006**
Natural yoghurt	2.50	1.40	2.60	1.45	0.478
Cottage cheese	3.48	1.14	3.48	1.17	0.931
Yellow cheese	3.59	1.21	3.63	1.35	0.293
Boys (*n* = 600)					
Milk	4.56	1.06	4.62	1.00	0.578
Fruit yoghurt	3.65	1.28	3.90	1.11	**0.044**
Natural yoghurt	2.30	1.38	2.58	1.38	**0.009**
Cottage cheese	3.42	1.17	3.48	1.21	0.439
Yellow cheese	3.53	1.37	3.76	1.19	0.061

BFP—body fat percentage; significant associations are highlighted in bold.

**Table 5:** Logistic regression analysis showing the association between dairy products consumption and excess adiposity.

Variables		B	SE	Wald	df	OR (95% CI)	*p*
Total sample (n = 1172)	Fruit yoghurt	0.183	0.051	12,722	1	1.20 (1.09–1.33)	**<0.001**
Girls (n = 572)	Milk	0.192	0.092	4323	1	1.21 (1.01–1.45)	**0.038**
	Fruit yoghurt	0.194	0.080	5938	1	1.21 (1.04–1.42)	**0.015**
Boys (n = 600)	Fruit yoghurt	0.156	0.070	4995	1	1.17 (1.02–1.34)	**0.025**
	Natural yoghurt	0.136	0.060	5144	1	1.15 (1.02–1.29)	**0.023**

df—degrees of freedom; OR (95% CI)—odds ratio with a 95% confidence interval; SE—standard error; significant associations are highlighted in bold.

## Data Availability

The data presented in this study are available on request from the corresponding author.
